# Optimal vaccination strategy for dengue transmission in Kupang city, Indonesia

**DOI:** 10.1016/j.heliyon.2020.e05345

**Published:** 2020-11-04

**Authors:** Meksianis Z. Ndii, Ananda R. Mage, Jakobis J. Messakh, Bertha S. Djahi

**Affiliations:** a Department of Mathematics, The University of Nusa Cendana, Kupang-NTT, Indonesia; b Department of Building Engineering Education, The University of Nusa Cendana, Kupang-NTT, Indonesia; c Department of Computer Science, The University of Nusa Cendana, Kupang-NTT, Indonesia

**Keywords:** Mathematics, Dengue, Model, Optimal control, Vaccination

## Abstract

Dengue is a public health problem with around 390 million cases annually and is caused by four distinct serotypes. Infection by one of the serotypes provides lifelong immunity to that serotype but have a higher chance of attracting the more dangerous forms of dengue in subsequent infections. Therefore, a perfect strategy against dengue is required. Dengue vaccine with 42-80% efficacy level has been licensed for the use in reducing disease transmission. However, this may increase the likelihood of obtaining the dangerous forms of dengue. In this paper, we have developed single and two-serotype dengue mathematical models to investigate the effects of vaccination on dengue transmission dynamics. The model is validated against dengue data from Kupang city, Indonesia. We investigate the effects of vaccination on seronegative and seropositive individuals and perform a global sensitivity analysis to determine the most influential parameters of the model. A sensitivity analysis suggests that the vaccination rate, the transmission probability and the biting rate have greater effects on the reduction of the proportion of dengue cases. Interestingly, with vaccine implementation, the mosquito-related parameters do not have significant impact on the reduction in the proportion of dengue cases. If the vaccination is implemented on seronegative individuals only, it may increase the likelihood of obtaining the severe dengue. To reduce the proportion of severe dengue cases, it is better to vaccinate seropositive individuals. In the context of Kupang City where the majority of individuals have been infected by at least one dengue serotype, the implementation of vaccination strategy is possible. However, understanding the serotype-specific differences is required to optimise the delivery of the intervention.

## 1 Introduction

Dengue is a public health problem and threatens two thirds of the world's population with around 390 million cases annually [1]. Nealon et al. [2] reported that the number of dengue cases is under-reported which may increase the possibility disease burden. Shepard et al. [3] estimated that around 58,40 million symptomatic cases with 13.586 fatal cases and the total annual cost of dengue was around US$ 8.9 billion.

Dengue is caused by four distinct serotypes where infections by one of the serotypes provide long-life immunity to the serotype they are infected with. Dengue is transmitted via a bite of *Aedes* mosquitoes particularly *Aedes aegeypti* which are mostly the main vector in most countries including Indonesia. When an infected mosquito bites humans, they have a chance to attract dengue. The infected human can then recover from dengue and have cross immunity for around six months [4]. After that, they are re-susceptible to the other dengue serotypes. The process repeats and the previously infected human may be re-infected by the other strain. The secondary infections may result in more dangerous forms of dengue, known as Dengue Hemorrhagic Fever (DHF) and Dengue Shock Syndrome (DSS) with the fatality rate of 20% without a proper treatment [5]. This is due to the effects of antibody-dependent enhancement (ADE). This means that strategies should be effective against all dengue serotypes.

A dengue vaccine with efficacy of around 54-77% has been approved for the use in reducing dengue transmission [6]. The vaccine effectiveness depends strongly on the age group and the transmission level [7]. Ferguson et al. [7] found that the vaccination benefits the entire population (seronegative and seropositive) in areas with high transmission levels. In areas with low and moderate transmission levels, an increase in the number of secondary infections may happen. Aguiar et al. [6] found that the use of dengue vaccine may increase the disease burden. Furthermore, Zheng et al. [8] performed cost-benefit analysis of the use of dengue vaccine and found that the routine vaccination would reduce the yearly illness cost by around 22-23% in Latin American and Asian countries.

A number of mathematical model have been formulated to investigate disease transmission dynamics [9] including the effects of dengue vaccine on its transmission dynamics. Aguiar et al. developed an age-structured mathematical model to assess the impact of vaccine on dengue transmission dynamics when it is implemented on different age group [6]. They found that the vaccine is effective if we vaccinate the seropositive individuals. The results are similar to that found by Ndii et al. [10] and Ferguson et al. [7]. Agusto et al. [11] developed a mathematical model and used the optimal control theory to analyse the optimal strategies for reducing the dengue transmission. They found that the use of insecticide and vaccine can reduce the same number of infections regardless of the weights on the costs. A dengue model with vaccination has been formulated and analysed by Rodrigues et al. [12]. They analysed the optimal control strategies to dengue and found that the reduction in the number of dengue cases depends on the type of vaccine and vaccination coverage. Aldila et al. [13] developed a age-structure dengue model and analysed several control parameters such as the treatment and the drop-out rates for children and adults. They found that the implementation of the treatment before the occurrence of the outbreak is more effective if the budget is limited. Shim [14] formulated and a mathematical model and analysed optimal dengue vaccination to reduce dengue transmission and focused on the vaccination on seropositive individuals. The results showed that at the early phase of epidemics, optimal dengue vaccination rates for seropositive individuals are highest. This implies that intense effort at the early phase of an epidemic is required. Pongsupum et al. formulated a mathematical model of dengue with vertical transmission and used an optimal control approach to examine the effects of vaccination, insecticides, and isolation. It was found that, although the administration of isolation and insecticides resulted a faster decline of the infected human population, a greater expense in the initial effort is required. On the other hand, the required vaccination effort is significantly less [15].

In this paper, we formulate single and two serotype dengue mathematical models model with vaccination and use the optimal control approach to determine the optimal vaccination strategy against dengue. We validate the model against 2016 dengue data in Kupang city, Indonesia. We perform a global sensitivity analysis to determine the most influential parameters of the model. Unlike the aforementioned work, in this paper, we consider seasonality using sinusoidal function. Seasonal forcing is included because the Kupang city has a strong dry and rainy seasons where the more mosquitoes exists in rainy season.

The remainder of the paper is organised as follows. Section 2 presents the single serotype dengue model with vaccination. This consists of model formulation, data and parameter estimation, sensitivity analysis, and optimal control analysis. Section 3 presents a two serotype dengue model, consisting of sensitivity analysis and optimal control analysis. Finally, the discussion and conclusions are presented.

## 2 Single serotype dengue mathematical model with vaccination

### 2.1 Model formulation

We present a mathematical model of dengue with vaccine. We adapt a single dengue model by Ndii et al. [16] for the case of vaccination. In the model, human population is divided into susceptible $\left(S_{H}\right)$, exposed $\left(E_{H}\right)$, infected $\left(I_{H}\right)$ and recovered $\left(R_{H}\right)$. We set the vaccine as a control and once the vaccine is implemented, the individuals move to recovered compartment. We take into account waning immunity. That is, the recovered individuals may loss immunity and become susceptible again. The mosquito population is divided into aquatic $\left(A_{N}\right)$, susceptible $\left(S_{N}\right)$, exposed $\left(E_{N}\right)$, and infected $\left(I_{N}\right)$. The model is governed by the following system of differential equation

(1) $$\frac{dS_{H}}{dt}=-b_{N}T_{N}LI_{N}S_{H}-\mu_{H}S_{H}-uS_{H}+\mu_{H}+\theta uR_{H},$$

(2) $$\frac{dE_{H}}{dt}=b_{N}T_{N}LI_{N}S_{H}-\gamma_{H}E_{H}-\mu_{H}E_{H},$$

(3) $$\frac{dI_{H}}{dt}=\gamma_{H}E_{H}-\sigma I_{H}-\mu_{H}I_{H},$$

(4) $$\frac{dR_{H}}{dt}=\sigma I_{H}+uS_{H}-\theta uR_{H}-\mu_{H}R_{H},$$

(5) $$\frac{dA_{N}}{dt}=\rho_{N}\frac{F_{N}}{2}(1-(A_{N}))-\left(\tau_{N}+\mu_{NA}\right)A_{N},$$

(6) $$\frac{dS_{N}}{dt}=\tau_{N}\frac{A_{N}}{2}-\left(b_{N}T_{N}I_{H}+\mu_{N}(t)\right)S_{N},$$

(7) $$\frac{dE_{N}}{dt}=b_{N}T_{N}I_{H}S_{N}-\left(\gamma_{N}+\mu_{N}(t)\right)E_{N},$$

(8) $$\frac{dI_{N}}{dt}=\gamma_{N}E_{N}-\mu_{N}(t)I_{N},$$

where

$$\mu_{N}(t)=\mu_{N0}\left(1-\eta \cos\left(\frac{2\pi (t+\omega )}{365}\right)\right).$$

The susceptible individuals are exposed after being bitten by infected mosquitoes at a rate $b_{N}T_{N}$, where $b_{N}$ is the biting rate and $T_{N}$ is the probability of successful transmission. The exposed individuals become infectious at a rate of $\gamma_{H}$ and then recovered at a rate of *σ*. The susceptible individuals are vaccinated at rate *u*. The parameter *θ* represents the waning immunity process. The mosquito population is produced after the male and female mosquitoes mate and produce offspring at a rate $\rho_{N}$ and its growth is limited by carrying capacity, K, which is governed by

(9) $$\rho_{N}\frac{F_{N}M_{N}}{F_{N}+M_{N}}\left(1-\frac{A_{N}}{K}\right).$$

We assume that the ratio of male and female mosquitoes is 1:1, and hence the $M_{N}=F_{N}$, and be normalised the equation by *K* we obtain

$$\rho_{N}\frac{F_{N}}{2}(1-A_{N}).$$

Susceptible mosquitoes become exposed to dengue after bitting infected human at a rate $b_{N}T_{N}$ and then become infectious at a rate $\gamma_{N}$. The infected mosquitoes remain infectious for the rest of their life.

### 2.2 Data and parameter estimation

In this part, we estimate the parameter values using 2016 weekly data of dengue cases in Kupang city, East Nusa Tenggara, Indonesia. The data has been obtained from The Health Office of Kupang City, East Nusa Tenggara Province. We use the model before the implementation of vaccination. We parameterise the model using ‘lsqnonlin’ function in MATLAB. There are four parameters to be estimated: $T_{N}$, $\gamma_{H}$, *η*, *ω*. The other parameters are obtained from the literature and are given in Table 1. The human death rate is taken to be 1/66.5 [17]. The vaccine efficacy is set to be 0.5 for the seronegative individuals and 0.77 for seropositive individuals [6], [18]. The period of cross immunity is taken to be 6 months [4], [14]. The rate of antibody-dependent enhancement is set to be 1.1 [19]. The average mosquito death rate and the reproduction rate are set to be 1/14 and 1.25, respectively [20], [16]. In addition, the biting rate and the aquatic mosquito death rate are 0.63 and 1/14 respectively. Detail of the parameter values and units are given in Table 1. As the model is formulated in the proportion, we divided the number of infection with 402286, which is the total population in Kupang city in 2016.

**Table 1:** Parameter descriptions, values and sources for the mathematical models.

Symbol	Description	Value	Unit	Source
*T*_*N*_	Transmission probability	0.22095	N/A	Fitted
*b*_*N*_	Biting rate	0.63	day${}^{\text{-1}}$	[32]
*ω*	Phase	47.772	day	Fitted
*μ*_*N*0_	Average adult mosquito death rate	1/14	day${}^{\text{-1}}$	[20]
*τ*_*N*_	Maturation rate	1/10	day${}^{\text{-1}}$	[20]
*σ*	Recovery rate	1/5	day${}^{\text{-1}}$	[33]
*η*	Seasonality amplitude	0.551253	N/A	Fitted
*ρ*_*N*_	Reproductive rate	1.25	day${}^{\text{-1}}$	[16]
*μ*_*NA*_	Aquatic death rate	1/14	day${}^{\text{-1}}$	[20]
*γ*_*N*_	Progression from exposed to infectious class (mosquitoes)	1/10	day${}^{\text{-1}}$	[34]
*γ*_*H*_	Progression from exposed to infectious class (human)	0.199999	day${}^{\text{-1}}$	Fitted
*L*	Ratio of carrying capacity in comparison to total human population	3	N/A	[34]
1/*α*	Cross immune period	182.5	day	[14], [4]
*ϵ*_1_	Vaccine efficacy for seronegative individuals	0.5	N/A	[6], [18]
*ϵ*_2_	Vaccine efficacy for seropositive individuals	0.77	N/A	[6], [18]
*ζ*	The rate of antibody-dependent enhancement	1.1	N/A	[19]
*μ*_*H*_	Human death rate	1/(66.5)	year${}^{\text{-1}}$	[17]
*u*_1_	Control/vaccination rate on seronegative individuals	[0 1]	day${}^{\text{-1}}$	Simulated
*u*_2_	Control/vaccination rate on seropositive individuals	[0 1]	day${}^{\text{-1}}$	Simulated

We minimise the sum of squared error which is given by

(10) $$SSE=\sum_{n=1}^{m}{\left(y_{n}-f_{n}(x)\right)}^{2}$$

where $y_{n}$ is the total proportion of human dengue cases up to week 52 and $f_{n}(x)$ is the total proportion of human dengue up to week *n* from model's simulation.

**Figure 1:**
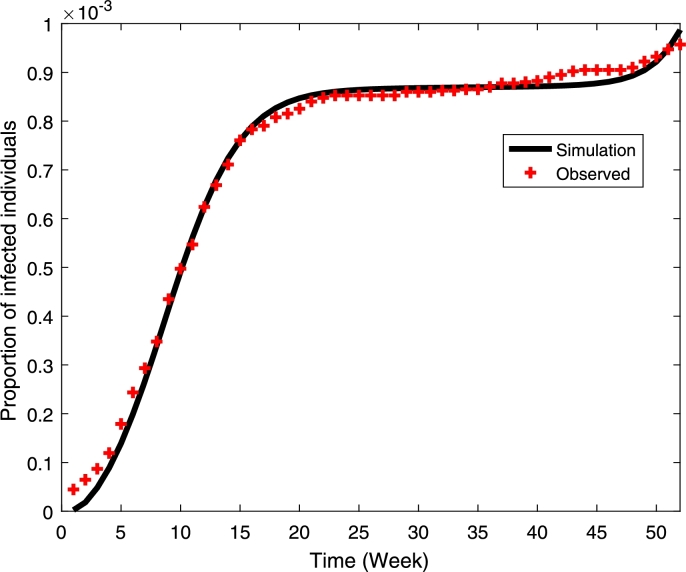
Plot of the model's simulation and the observed dengue cases in Kupang City, East Nusa Tenggara, Indonesia.

The fitted values are $T_{N}=0.22095$ (CI: 0.21875, 0.22315), η=0.551253 (CI: 0.42683, 0.67567), ω=47.772 (CI: 45.45158, 50.09330), $\gamma_{H}=0.199999$ (CI: 0.01069, 0.38930) with the residual norm of $2.092210406871739\times 10^{-8}$ and the plot of simulated and observed values is given in Fig. 1. It shows that the model fits well with the data. We also estimate using Multistart in Matlab and found the similar results and hence it found the global optimum.

### 2.3 Sensitivity analysis

In this part, we perform a global sensitivity analysis to determine the important parameters of the model. We use the combination of Latin Hypercube Sampling (LHS) in conjunction with Partial Rank Correlation Coefficient (PRCC) to assess the influential parameters of the model.

Latin Hypercube Sampling is a stratified sampling without replacement technique that divides the parameter ranges into *N* equal probability intervals and samples are randomly drawn from each interval [21], [22]. PRCC measures the nonlinear but monotonic relationship between inputs and outputs [21], [22]. In our analysis, the inputs are the parameters and the model's outcomes are the cumulative number of infected individuals for single serotype dengue model, and primary, secondary and overall infections for two serotype dengue model.

**Figure 2:**
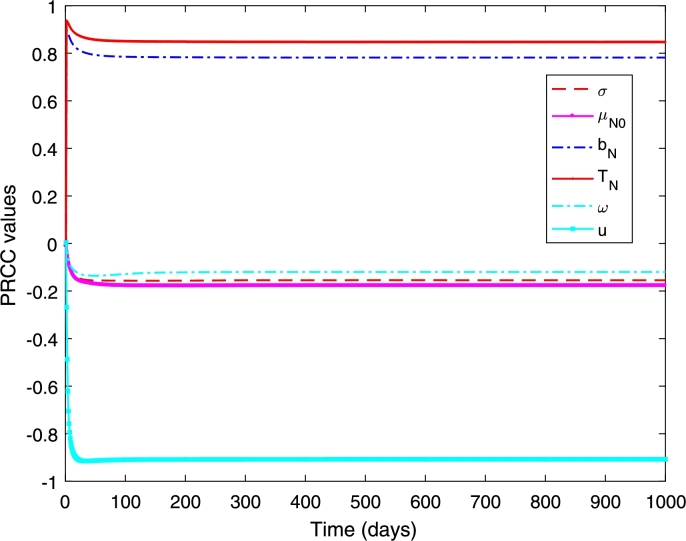
PRCC values when measured against the increasing proportion of the dengue infections.

The outcome of interest is the increasing number of infected individuals which is

(11) $$C_{p}(t)=\int_{0}^{T}(\gamma_{H}E_{H})dt,$$

where *T* is the final time of interest. Fig. 2 shows that the transmission probability $\left(T_{N}\right)$, the biting rate $\left(b_{N}\right)$, and the vaccination rate (*u*) are the most influential parameters. The first two have positive relationship and the last one has negative relationship. This indicates that when the values of $T_{N}$ and $b_{N}$ increase, the total proportion of dengue infection also increases. On the other hand, if the vaccination rate (*u*) increases, the total proportion of infection decreases. This indicates that increasing the vaccination rate and reducing the transmission probability and the biting rate can minimise the proportion of dengue infections. Furthermore, these three parameters strongly influential since the early period of epidemics and remain influential and govern the disease transmission dynamics until the end period. The remain parameters (*ω*, $\mu_{N0}$, *σ*) have negative relationships although their influence is not as strong as the other three parameters. For example, if the average death rate of mosquitoes is high (short mosquito lifespan), the proportion of dengue infections has declined.

### 2.4 Optimal vaccination strategy in the presence of single serotype dengue

The aim of this study is to study the optimal vaccination strategy. Let the $W_{1}$, $W_{2}$ and $W_{3}$ are the weight constant that represent the cost of treatment of exposed and infected individuals, and vaccination, respectively. We aim to minimise the proportion of infected individuals and cost of vaccination. We define an objective the objective functional to be minimised as

(12) $$J(u)=\int_{0}^{t_{f}}\left(W_{1}E_{H}(t)+W_{2}I_{H}(t)+W_{3}S_{H}(t)u^{2}(t)\right)dt$$

subjected to the Model (1)–(8).

We use the quadratic terms in the control variables to represent the nonlinear cost in the implementation of the control. It is generally believed that there is no linear relationship between effects of intervention and the cost of intervention [23], [11] and hence the quadratic costs have been commonly used [11], [13], [23], [24], [25], [26]. This approach is rather conventional in the optimal control problems of the epidemiological modelling and this simplifies the mathematical analysis [23], [26]. The use of linear term in the cost function leads to bang-bang control [23], [27], [28]. The other work that used other terms in the control variables can be found in [29], [30], which can be considered for the future investigation. Explanations of the optimal control approach in the biological problems can be found in [28]. The Hamiltonian function is given by

(13) $$H=W_{1}E_{H}(t)+W_{2}I_{H}(t)+W_{3}S_{H}(t)u^{2}+\lambda_{S_{H}}\left(-b_{N}T_{N}LI_{N}S_{H}-\mu_{H}S_{H}-uS_{H}+\mu_{H}+\theta uR_{H}\right)+\lambda_{E_{H}}\left(b_{N}T_{N}LI_{N}S_{H}-\gamma_{H}E_{H}-\mu_{H}E_{H}\right)+\lambda_{I_{H}}\left(\gamma_{H}E_{H}-\sigma I_{H}-\mu_{H}I_{H}\right)+\lambda_{R_{H}}\left(\sigma I_{H}+uS_{H}-\theta uR_{H}-\mu_{H}R_{H}\right)+\lambda_{A_{N}}\left(\rho_{N}\frac{F_{N}}{2}(1-(A_{N}))-\left(\tau_{N}+\mu_{NA}\right)A_{N}\right)+\lambda_{S_{N}}\left(\tau_{N}\frac{A_{N}}{2}-\left(b_{N}T_{N}I_{H}+\mu_{N}(t)\right)S_{N}\right)+\lambda_{E_{N}}\left(b_{N}T_{N}I_{H}S_{N}-\left(\gamma_{N}+\mu_{N}(t)\right)E_{N}\right)+\lambda_{I_{N}}\left(\gamma_{N}E_{N}-\mu_{N}(t)I_{N}\right),$$

where $\lambda_{S_{H}}$, $\lambda_{E_{H}}$, $\lambda_{I_{H}}$, $\lambda_{R_{H}}$, $\lambda_{A_{N}}$, $\lambda_{S_{N}}$, $\lambda_{E_{N}}$, $\lambda_{I_{N}}$ are the associated adjoints for the states $S_{H}$, $E_{H}$, $I_{H}$, $R_{H}$, $A_{N}$, $S_{N}$, $E_{N}$, $I_{N}$, respectively.

In the hamiltonian function, *H*, each adjoint function multiplies the right-hand side of the differential equation of its corresponding state function. The first term in *H* is from the integrand of the objective functional.

Theorem 2.1 *There exist optimal controls,* $u^{⁎}$ *and state solutions of the corresponding system that maximise* J(u) *over the set U. Then there exist adjoint variables* $\lambda_{l}$ *satisfying*

$$\frac{d\lambda_{l}}{dt}=-\frac{\partial H}{\partial l}$$

*where* $l=S_{H},E_{H},I_{H},R_{H},A_{N},S_{N},E_{N},I_{N}$ *and with transversality condition* $\lambda_{i}(t_{f})=0$ *The optimality conditions are given as*

$$\frac{\partial H}{\partial u}=0.$$

*Furthermore, the control* $u^{⁎}$ *is given as*

(14) $$u^{⁎}(t)=\min\left\{1,\max\left[0,\frac{1}{2}\frac{\theta R_{H}(\lambda_{R_{H}}-\lambda_{S_{H}})-S_{H}(\lambda_{R_{H}}-\lambda_{S_{H}})}{W_{3}S_{H}}\right]\right\}$$

Proof 2.1 The differential equations of the adjoint variables are obtained by the differentiation of the Hamiltonian function, $\frac{d\lambda_{i}}{dt}=-\frac{\partial H}{\partial i}$. Thus, the adjoint system is given by

$$\frac{d\lambda_{S_{H}}}{dt}=-W_{3}u^{2}-\lambda_{E_{H}}b_{N}T_{N}LI_{N}-\lambda_{R_{H}}u-\lambda_{S_{H}}(-Lb_{N}T_{N}I_{N}-u-\mu_{H}),\frac{d\lambda_{E_{H}}}{dt}=-W_{1}-\lambda_{E_{H}}(-\gamma_{H}-\mu_{H})-\lambda_{I_{H}}\gamma_{H},\frac{d\lambda_{I_{H}}}{dt}=-W_{2}-\lambda_{E_{N}}b_{N}T_{N}S_{N}-\lambda_{R_{H}}\sigma +\lambda_{S_{N}}b_{N}T_{N}S_{N}-\lambda_{I_{H}}(-\sigma -\mu_{H}),\frac{d\lambda_{R_{H}}}{dt}=-\lambda_{R_{H}}(-\theta u-\mu_{H})-\lambda_{S_{H}}\theta u\;\frac{d\lambda_{A_{N}}}{dt}=-\lambda_{A_{N}}(-\frac{1}{2}\rho_{N}F_{N}-\tau_{N}-\mu_{NA})-\frac{1}{2}\lambda_{S_{N}}\tau_{N},\frac{d\lambda_{S_{N}}}{dt}=-\lambda_{E_{N}}b_{N}T_{N}I_{H}-\lambda_{S_{N}}(-b_{N}T_{N}I_{H}-\mu_{N}(t)),\frac{d\lambda_{E_{N}}}{dt}=-\lambda_{E_{N}}(-\gamma_{N}-\mu_{N}(t))-\lambda_{I_{N}}\gamma_{N},\frac{d\lambda_{I_{N}}}{dt}=-\lambda_{E_{H}}Lb_{N}S_{H}T_{N}+\lambda_{S_{H}}Lb_{N}S_{H}T_{N}+\lambda_{I_{N}}\mu_{N}(t)),\text{where}\;\mu_{N}(t)=\mu_{N0}\left(1-\eta \cos\left(\frac{2\pi (t+\omega )}{365}\right)\right).$$

Furthermore, differentiating the Hamiltonian function with respect to the control variables *u* to obtain

$$\frac{\partial H}{\partial u}=2W_{3}uS_{H}+\lambda_{R_{H}}(-\theta R_{H}+S_{H})+\lambda_{S_{H}}(\theta R_{H}-S_{H})=0.$$

Solving for $u^{⁎}$, we obtain

$$u^{⁎}=\frac{1}{2}\frac{\theta R_{H}(\lambda_{R_{H}}-\lambda_{S_{H}})-S_{H}(\lambda_{R_{H}}-\lambda_{S_{H}})}{W_{3}S_{H}}.$$

Using the bounds of the control, we obtain the characterisation given in Equation (14).

### 2.5 Numerical simulations

The optimality system is solved numerically using forward-backward sweep numerical method [28], [31], [24]. First, the initial guess of the optimal control is determined. Next, the state variables are solved forward in time which is then substituted into the adjoint equations. Furthermore, the adjoint equations are solved backward in time using ode45 in MATLAB. The state and adjoint values are used to update the controls. This process is repeated until the state, adjoint and control values converge.

**Figure 3:**
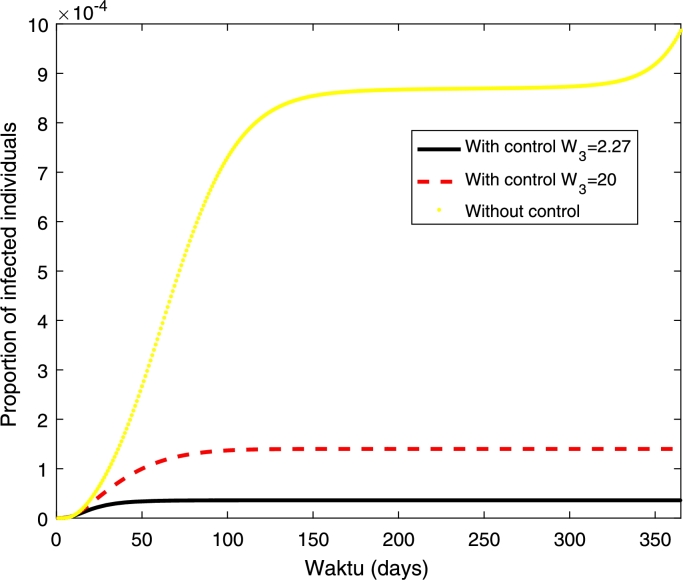
Numerical simulations with and without control. The cost of vaccination (*W*_3_) are 2.27 and 20 as given in legend, *W*_1_ = *W*_2_ = 380.

**Figure 4:**
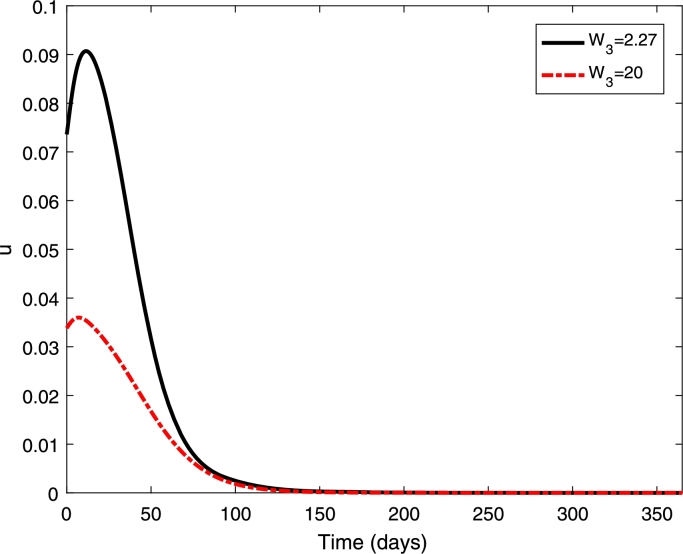
Control profiles (*u*) with different cost of vaccination *W*_3_ = 2.27 and 20.

In the numerical simulations, we use the following initial conditions $S_{H}(0)=0.999955255713597$, $E_{H}(0)=0$, $I_{H}(0)=0.000044744286403$, $R_{H}(0)=0$, $A_{N}(0)=0.791111869731644$, $S_{N}(0)=1.031725009589116$, $E_{N}(0)=0$, $I_{N}(0)=0$. The set of initial condition is found by running the model to stable state before dengue is introduced into the population. In the model, the mosquito population is normalised to carrying capacity of the aquatic mosquitoes. The initial condition for susceptible mosquitoes is greater than one which means that the population is higher than carrying capacity of aquatic mosquitoes. Furthermore, we start the simulation from January where the mosquito population is at high level. The other parameter values are taken from the literature. Zeng et al. found that the treatment cost for hospitalised case is US$ 380 and the vaccine delivery cost is US$ 2.27 [8]. We also simulate the case where the vaccine cost is expensive by assuming the vaccine cost of US$ 20. Hence, in the simulation, we use $W_{1}=W_{2}=380$ and $W_{3}=2.27\;\text{and}\;20$. The numerical solution and the control profile are given in Figs. 3 and 4.

Fig. 3 shows that the proportion of infected individuals has been reduced with the implementation of vaccination strategy where higher reduction can be obtained if the cost of vaccination is cheaper. Furthermore, higher control (vaccination) rate can be obtained if the cost of vaccination is cheaper. Furthermore, it shows that a higher control rate should be given in the early period before it has been reduced at the end of the period (see Fig. 4).

## 3 Two serotype dengue model with vaccination

### 3.1 Mathematical formulation

In this section, we present the two-serotype dengue model with vaccination by extending the single serotype dengue model. We also include seasonality on the mosquito death rate. We consider the vaccination on seronegative and seropositive individuals. The human population is divided into the fully susceptible $\left(S_{H}\right)$, seronegative vaccinated $\left(V_{H}\right)$, primary exposed $\left(E_{H}^{k}\right)$, primary infected $\left(I_{H}^{k}\right)$, temporary recovered $\left(R_{H}^{k}\right)$, susceptible to the other strain that has not been previously infected with $\left(S_{H}^{k}\right)$, seropositive vaccinated $\left(V_{H}^{k}\right)$, secondary exposed $\left(X_{H}^{k}\right)$, secondary infected $\left(Y_{H}^{k}\right)$, and fully recovered (*Z*) individuals. The mosquito population is divided into the aquatic $\left(A_{N}\right)$, susceptible $\left(S_{N}\right)$, the exposed $\left(E_{N}^{k}\right)$, and the infected $\left(I_{N}^{k}\right)$ mosquitoes. The superscripts k=1,2 is to denote the dengue serotype. The model is governed by the following system of differential equations.

The vaccinated seronegative individuals move the vaccinated compartment $\left(V_{H}\right)$ at a rate $u_{1}$ and they become infected after being bitten by infected mosquitoes and the vaccine lose its efficacy at a rate $b_{N}T_{N}L(1-\epsilon_{1})$. We also consider the effects of antibody-dependent enhancement (ADE). When the susceptible mosquitoes bite secondary infected individuals, the rate of transmission is higher with the rate *ζ*. The vaccination is implemented to seropositive individuals ($R_{H}^{k}$ and $S_{H}^{k}$).

The model is governed by the following system of differential equations.

(15) $$\frac{dS_{H}}{dt}=\mu_{h}-Lb_{N}T_{N}S_{H}\sum_{k=1}^{2}I_{N}^{k}-u_{1}S_{H}-\mu_{H}S_{H},$$

(16) $$\frac{dV_{H}}{dt}=u_{1}S_{H}-L(1-\epsilon_{1})b_{N}T_{N}V_{H}\sum_{k=1}^{2}I_{N}^{k}-\mu_{H}V_{H},$$

(17) $$\frac{dE_{H}^{k}}{dt}=Lb_{N}T_{N}S_{H}I_{N}^{k}-\gamma_{H}E_{H}^{k}-\mu_{H}E_{H}^{k},$$

(18) $$\frac{dI_{H}^{k}}{dt}=\gamma_{H}E_{H}^{k}-(\sigma +\mu_{H})I_{H}^{k},$$

(19) $$\frac{dR_{H}^{k}}{dt}=\sigma I_{H}^{k}-(\alpha +u_{2}+\mu_{H})R_{H}^{k},$$

(20) $$\frac{dS_{H}^{k}}{dt}=\alpha R_{H}^{k}-Lb_{N}T_{N}I_{N}^{j}S_{H}^{k}-(u_{2}+\mu_{H})S_{H}^{k},\;j\neq k,$$

(21) $$\frac{dV_{H}^{k}}{dt}=u_{2}(S_{H}^{k}+R_{H}^{k})-Lb_{N}T_{N}(1-\epsilon_{2})\beta_{h}I_{N}^{j}V_{H}^{k}-\mu_{h}V_{H}^{k},\;j\neq k,$$

(22) $$\frac{dX_{H}^{k}}{dt}=Lb_{N}T_{N}I_{N}^{k}((1-\epsilon_{1})V_{H}+(1-\epsilon_{2})V_{H}^{j}+S_{H}^{j})$$

(23) $$\;-\gamma_{H}X_{H}^{k}-\mu_{H}X_{H}^{k},\;j\neq k,\frac{dY_{H}^{k}}{dt}=\gamma_{H}X_{H}^{k}-(\sigma +\mu_{H})Y_{H}^{k},$$

(24) $$\frac{dZ}{dt}=\sigma \sum_{k=1}^{2}Y_{H}^{k}-\mu_{H}Z,$$

(25) $$\frac{dA_{N}}{dt}=\rho_{N}\frac{F_{N}}{2}(1-(A_{N}))-\left(\tau_{N}+\mu_{NA}\right)A_{N},$$

(26) $$\frac{dS_{N}}{dt}=\frac{\tau_{N}A_{N}}{2}-b_{N}T_{N}\sum_{k=1}^{2}I_{H}^{k}S_{N}-\zeta b_{N}T_{N}\sum_{k=1}^{2}X_{H}^{k}S_{N}-\mu_{N}(t)S_{N},$$

(27) $$\frac{dE_{N}^{k}}{dt}=b_{N}T_{N}I_{H}^{k}S_{N}+\zeta b_{N}T_{N}X_{H}^{k}S_{H}^{k}-(\gamma_{N}+\mu_{N}(t))E_{N}^{k},$$

(28) $$\frac{dI_{N}^{k}}{dt}=\gamma_{N}E_{N}^{k}-\mu_{N}(t)I_{N}^{k},$$

where $\mu_{N}(t)=\mu_{N0}\left(1-\eta \cos\left(\frac{2\pi (t+\omega )}{365}\right)\right)$.

### 3.2 Sensitivity analysis

Here we perform a global sensitivity analysis to determine the most influential parameters of the model. We measure against the increasing proportion of primary and secondary infections. The increasing proportion of the primary and secondary infection are

$$C_{p}(t)=\int_{0}^{T}\left(\gamma_{H}\sum_{k=1}^{2}E_{H}^{k}\right)dt,C_{s}(t)=\int_{0}^{T}\left(\gamma_{H}\sum_{k=1}^{2}X_{H}^{k}\right)dt,$$

where $C_{p}(t)$ and $C_{s}(t)$ are the total proportion of primary and secondary infections, respectively, and *T* is the end time of interest. The total proportion of dengue infections are the sum of $C_{p}(t)$ and $C_{s}(t)$.

**Figure 5:**
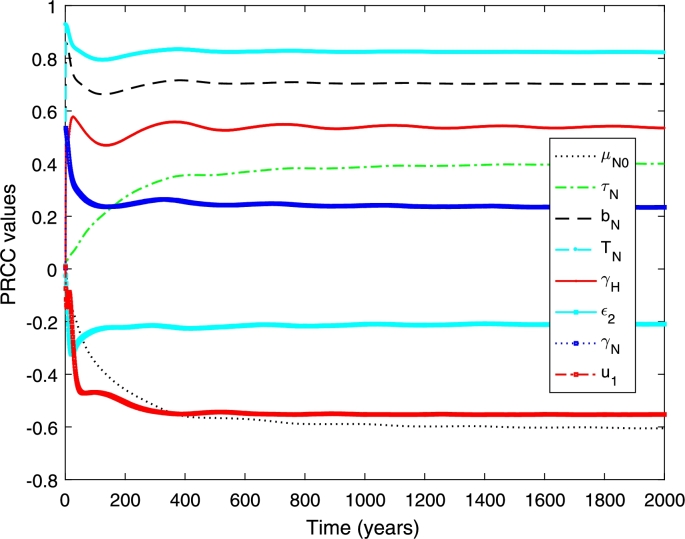
PRCC values measured against increasing number of total infection.

**Figure 6:**
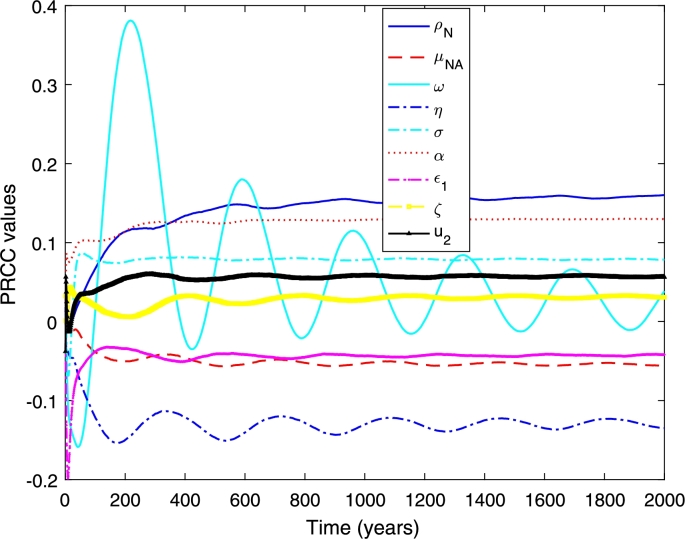
PRCC values measured against increasing number of total infection.

Figs. 5 and 6 show the PRCC values when measured against the total proportion of infected individuals. We found that the transmission probability $\left(T_{N}\right)$, the biting rate $\left(b_{N}\right)$, and the vaccination rate on seronegative individuals $\left(u_{1}\right)$ and the average death rate ($\mu_{N0}$) are the most influential parameters (see Fig. 5). The first two have positive relationship and the latter have negative relationship. This means that increasing the vaccination rate on seronegative individuals is required to reduce the proportion of overall dengue infections. Furthermore, the transmission probability and the biting rate needs to be reduced to minimise the proportion of overall dengue cases. For the other parameters (see Fig. 6), the phase shift strongly fluctuates in the early period of epidemics and between positive and negative relationships. The degree of seasonality (*η*) has positive relationship with the increasing proportion of overall infections. On the other hand, the reproduction rate $(\rho_{N}$) has negative relationship.

### 3.3 Optimal vaccination strategy in the presence of two dengue serotypes

In this section, we present the optimal control problem for two serotype dengue model. We aim to minimise the proportion of infected individuals and minimise the cost of vaccination. We define the objective functional to be minimised as

(29) $$J(u_{1},u_{2})=\int_{0}^{t_{f}}(W_{1}\sum_{k=1}^{2}(E_{H}^{k}(t))+W_{2}\sum_{k=1}^{2}(I_{H}^{k}(t))+W_{3}\sum_{k=1}^{2}(X_{H}^{k}(t))+W_{4}\sum_{k=1}^{2}(Y_{H}^{k}(t))+W_{5}S_{H}(t)u_{1}^{2}(t)+W_{6}\sum_{k=1}^{2}(R_{H}^{k}(t)+S_{H}^{k}(t))u_{2}^{2}(t))dt,$$

where the control effect is modelled by quadratic terms in $u_{1}$ and $u_{2}$. We model the control efforts by quadratic terms in order to incorporate the nonlinear cost in the implementation of controls as explained in the previous section. The objective is to minimize the proportion of primary and secondary infections and the cost of implementing the vaccine on seronegative and seropositive individuals by using possible minimal control variables $u_{i}$ for i=1,2. In the objective function, $W_{1}$, $W_{2}$ represent the weight constants of the exposed and infected primary infections, respectively, $W_{3}$ and $W_{4}$ represent the weight constants of the exposed and infected secondary infections, $W_{5}$ and $W_{6}$ represent the cost of implementing vaccine on seronegative and seropositive individuals, respectively. The first four sums in the objective function are the cost due to primary and secondary infections, respectively. The remaining terms are the cost due to implementing vaccine on primary and secondary infections.

Let $l=S_{H}$, $V_{H}$, $E_{H}^{k}$, $I_{H}^{k}$, $R_{H}^{k}$, $S_{H}^{k}$, $V_{H}^{k}$, $X_{H}^{k}$, $Y_{H}^{k}$, *Z*, $A_{N}$, $S_{N}$, $E_{N}^{k}$, $I_{N}^{k}$ where k=1,2. The Hamiltonian function is the following

(30) $$H=W_{1}\sum_{k=1}^{2}(E_{H}^{k}(t))+W_{2}\sum_{k=1}^{2}(I_{H}^{k}(t))+W_{3}\sum_{k=1}^{2}(X_{H}^{k}(t))\;+W_{4}\sum_{k=1}^{2}(Y_{H}^{k}(t))+W_{5}S_{H}(t)u_{1}^{2}\;\;+W_{6}\sum_{k=1}^{2}(S_{H}^{k}(t)+R_{H}^{k}(t))u_{2}^{2}+\sum \lambda_{l}\frac{dl}{dt}.$$

Theorem 3.1 *There exist optimal controls,* $u_{1}^{⁎}$ *and* $u_{2}^{⁎}$ *and state solutions of the corresponding system that maximise* $J(u_{1},u_{2})$ *over the set U. Then there exist adjoint variables* $\lambda_{l}$ *satisfying*

$$\frac{d\lambda_{l}}{dt}=-\frac{\partial H}{\partial l}$$

*with transversality condition* $\lambda_{l}(t_{f})=0$ *The optimality conditions are given as*

$$\frac{\partial H}{\partial u_{j}}=0,\;j=1,2.$$

*Furthermore, the controls* $u_{1}^{⁎}$ *and* $u_{2}^{⁎}$ *is given as*

(31) $$u_{1}^{⁎}(t)=\min\left\{1,\max\left[0,\frac{1}{2}\frac{\left(\lambda_{S_{H}}-\lambda_{V_{H}}\right)}{W_{5}}\right]\right\}u_{2}^{⁎}(t)=\min\left\{1,\max\left[0,\overset{ˆ}{u_{2}}\right]\right\}$$

*where* $\overset{ˆ}{u_{2}}=\frac{R_{H}^{1}(\lambda_{R_{H}^{1}}-\lambda_{V_{H}^{1}})+R_{H}^{2}(\lambda_{R_{H}^{2}}-\lambda_{V_{2}})+S_{H}^{1}(\lambda_{S_{H}^{1}}-\lambda_{V_{H}^{1}})+S_{H}^{2}(\lambda_{S_{H}^{2}}-\lambda_{V_{H}^{2}})}{2W_{6}\sum_{k=1}^{2}(S_{H}^{k}(t)+R_{H}^{k}(t))}$

Proof 3.1 The differential equations of the adjoint variables are obtained by the differentiation of the Hamiltonian function, $\frac{d\lambda_{l}}{dt}=-\frac{\partial H}{\partial l}$. Thus, the adjoint system is given by

$$\frac{d\lambda_{S_{H}}}{dt}=-W_{5}u_{1}^{2}-\lambda_{S_{H}}\left(-b_{N}T_{N}L(I_{N}^{1}+I_{N}^{2})-\mu_{H}-u_{1}\right)-\lambda_{V_{H}}u_{1}-\lambda_{E_{H}^{1}}b_{N}T_{N}LI_{N}^{1}-\lambda_{E_{H}^{2}}b_{N}T_{N}LI_{N}^{2},\frac{d\lambda_{V_{H}}}{dt}=-\lambda_{V_{H}}\left(-(1-\epsilon_{1})b_{N}T_{N}L(I_{N}^{1}+I_{N}^{2})-\mu_{H}\right)-\lambda_{X_{H}^{1}}b_{N}T_{N}L(1-\epsilon_{1})I_{N}^{1}-\lambda_{X_{H}^{2}}(1-\epsilon_{1})b_{N}T_{N}I_{N}^{2},\frac{d\lambda_{E_{H}^{1}}}{dt}=-W_{1}-\lambda_{I_{H}^{1}}\gamma_{H}-\lambda_{E_{H}^{1}}(-\gamma_{H}-\mu_{H}),\frac{d\lambda_{E_{H}^{2}}}{dt}=-W_{1}-\lambda_{I_{H}^{2}}\gamma_{H}-\lambda_{E_{H}^{2}}(-\gamma_{H}-\mu_{H}),\frac{d\lambda_{I_{H}^{1}}}{dt}=-W_{2}-\lambda_{E_{N}^{1}}b_{N}T_{N}S_{N}+\lambda_{S_{N}}b_{N}T_{N}S_{N}-\lambda_{R_{H}^{1}}\sigma -\lambda_{I_{H}^{1}}(-\sigma -\mu_{H}),\frac{d\lambda_{I_{H}^{2}}}{dt}=-W_{2}-\lambda_{E_{N}^{2}}b_{N}T_{N}S_{N}+\lambda_{S_{N}}b_{N}T_{N}S_{N}-\lambda_{R_{H}^{2}}\sigma -\lambda_{I_{H}^{2}}(-\sigma -\mu_{H}),\frac{d\lambda_{R_{H}^{1}}}{dt}=-W_{6}u_{2}^{2}-\lambda_{V_{H}^{1}}u_{2}-\lambda_{S_{H}^{1}}\alpha -\lambda_{R_{H}^{1}}(-\alpha -u_{2}-\mu_{H}),\frac{d\lambda_{R_{H}^{2}}}{dt}=-W_{6}u_{2}^{2}-\lambda_{V_{H}^{2}}u_{2}-\lambda_{S_{H}^{2}}\alpha -\lambda_{R_{H}^{2}}(-\alpha -u_{2}-\mu_{H}),\frac{d\lambda_{S_{H}^{1}}}{dt}=-W_{6}u_{2}^{2}-\lambda_{X_{H}^{2}}b_{N}T_{N}LI_{N}^{2}-\lambda_{V_{H}^{1}}u_{2}-\lambda_{S_{H}^{1}}(-LT_{N}I_{N}^{2}b_{N}-u_{2}-\mu_{H}),\frac{d\lambda_{S_{H}^{2}}}{dt}=-W_{6}u_{2}^{2}-\lambda_{X_{H}^{1}}b_{N}T_{N}LI_{N}^{1}-\lambda_{V_{H}^{2}}u_{2}-\lambda_{S_{H}^{2}}(-LT_{N}I_{N}^{1}b_{N}-u_{2}-\mu_{H}),\frac{d\lambda_{V_{H}^{1}}}{dt}=-\lambda_{X_{H}^{2}}b_{N}T_{N}LI_{N}^{2}(1-\epsilon_{2})-\lambda_{V_{H}^{1}}(-b_{N}T_{N}LI_{N}^{2}(1-\epsilon_{2})-\mu_{H})\frac{d\lambda_{V_{H}^{2}}}{dt}=-\lambda_{X_{H}^{1}}b_{N}T_{N}LI_{N}^{1}(1-\epsilon_{2})-\lambda_{V_{H}2}(-b_{N}T_{N}LI_{N}^{1}(1-\epsilon_{2})-\mu_{H}),\frac{d\lambda_{X_{H}^{1}}}{dt}=-W_{3}-\lambda_{Y_{H}^{1}}\gamma_{H}-\lambda_{X_{H}^{1}}(-\gamma_{H}-\mu_{H}),\frac{d\lambda_{X_{H}^{2}}}{dt}=-W_{3}-\lambda_{Y_{H}^{2}}\gamma_{H}-\lambda_{X_{H}^{2}}(-\gamma_{H}-\mu_{H}),$$

$$\frac{d\lambda_{Y_{H}^{1}}}{dt}=-W_{4}-\lambda_{E_{N}^{1}}\zeta b_{N}T_{N}S_{N}+\lambda_{S_{N}}\zeta b_{N}T_{N}S_{N}-\lambda_{Z}\sigma -\lambda_{Y_{H}^{1}}(-\sigma -\mu_{H}),\frac{d\lambda_{Y_{H}^{2}}}{dt}=-W_{4}-\lambda_{E_{N}^{2}}\zeta b_{N}T_{N}S_{N}+\lambda_{S_{N}}\zeta b_{N}T_{N}S_{N}-\lambda_{Z}\sigma -\lambda_{Y_{H}^{2}}(-\sigma -\mu_{H}),\frac{d\lambda_{Z}}{dt}=\lambda_{Z}\mu_{h},\frac{d\lambda_{A_{N}}}{dt}=-\frac{1}{2}\lambda_{S_{N}}\tau_{N}-\lambda_{A_{N}}(-\frac{1}{2}\rho_{N}(S_{N}+E_{N}^{1}+E_{N}^{2}+I_{N}^{1}+I_{N}^{2})-\tau_{N}-\mu_{NA}),\frac{d\lambda_{S_{N}}}{dt}=-\lambda_{E_{N}^{2}}(b_{N}T_{N}Y_{H}^{2}\zeta +b_{N}T_{N}I_{H}^{2})-\lambda_{E_{N}^{1}}(b_{N}T_{N}\zeta Y_{H}^{1}+I_{H}^{1}T_{N}b_{N})-\lambda_{S_{N}}(-b_{N}T_{N}(I_{H}^{1}+I_{H}^{2})-\zeta b_{N}T_{N}(Y_{H}^{1}+Y_{H}^{2})-\mu_{N})-\frac{1}{2}\lambda_{A_{N}}\rho_{N}(1-A_{N}),\frac{d\lambda_{E_{N}^{1}}}{dt}=-\lambda_{I_{N}^{1}}\gamma_{N}-\lambda_{E_{N}^{1}}(-\gamma_{N}-\mu_{N})-\frac{1}{2}\lambda_{A_{N}}\rho_{N}(1-A_{N}),\frac{d\lambda_{E_{N}^{2}}}{dt}=-\lambda_{I_{N}^{2}}\gamma_{N}-\lambda_{E_{N}^{2}}(-\gamma_{N}-\mu_{N})-\frac{1}{2}\lambda_{A_{N}}\rho_{N}(1-A_{N}),\frac{d\lambda_{I_{N}^{1}}}{dt}=-\frac{1}{2}\lambda_{A_{N}}\rho_{N}(1-A_{N})-\lambda_{X_{H}^{1}}b_{N}T_{N}L(S_{H}^{2}+(1-\epsilon_{2})V_{H}^{2}+(1-\epsilon_{1})V_{H})+\lambda_{V_{H}^{2}}b_{N}T_{N}L(1-\epsilon_{2})V_{H}^{2}+\lambda_{S_{H}^{2}}b_{N}T_{N}LS_{H}^{2}-\lambda_{E_{H}^{1}}b_{N}T_{N}LS_{H}+\lambda_{V_{H}}(1-\epsilon_{1})b_{N}T_{N}LV_{H}+\lambda_{S_{H}}b_{N}T_{N}LS_{H},\frac{d\lambda_{I_{N}^{2}}}{dt}=-\frac{1}{2}\lambda_{A_{N}}\rho_{N}(1-A_{N})-\lambda_{X_{H}^{2}}b_{N}T_{N}L(S_{H}^{1}+(1-\epsilon_{2})V_{H}^{1}+(1-\epsilon_{1})V_{H})+\lambda_{V_{H}^{1}}b_{N}T_{N}L(1-\epsilon_{2})V_{H}^{1}+\lambda_{S_{H}^{1}}LT_{N}b_{N}S_{H}^{1}-\lambda_{E_{H}^{2}}b_{N}T_{N}LS_{H}+\lambda_{V_{H}}(1-\epsilon_{1})b_{N}T_{N}LV_{H}+\lambda_{S_{H}}b_{N}T_{N}LS_{H}.$$

Furthermore, differentiating the Hamiltonian function with respect to the control variables $\left(u_{1},u_{2}\right)$ to obtain

$$\frac{\partial H}{\partial u_{1}}=-S_{H}\lambda_{S_{H}}+S_{H}\lambda_{V_{H}}+2W_{5}S_{H}u_{1}=0,\frac{\partial H}{\partial u_{2}}=2W_{6}u_{2}\sum_{k=1}^{2}(S_{H}^{k}(t)+R_{H}^{k}(t))+\lambda_{V_{H}^{2}}(R_{H}^{2}+S_{H}^{2})+\lambda_{V_{H}^{1}}(R_{H}^{1}+S_{H}^{1})\;-\lambda_{S_{H}^{2}}S_{H}^{2}-\lambda_{S_{H}^{1}}S_{H}^{1}-\lambda_{R_{H}^{2}}R_{H}^{2}-\lambda_{R_{H}^{1}}R_{H}^{1}=0$$

Solving for $u_{1}^{⁎}$ and $u_{2}^{⁎}$, we obtain

$$u_{1}^{⁎}=\frac{1}{2}\frac{\left(\lambda_{S_{H}}-\lambda_{V_{H}}\right)}{W_{5}},u_{2}^{⁎}=\frac{R_{H}^{1}(\lambda_{R_{H}^{1}}-\lambda_{V_{H}^{1}})+R_{H}^{2}(\lambda_{R_{H}^{2}}-\lambda_{V_{H}^{2}})+S_{H}^{1}(\lambda_{S_{H}^{1}}-\lambda_{V_{H}^{1}})+S_{H}^{2}(\lambda_{S_{H}^{2}}-\lambda_{V_{H}^{2}})}{2W_{6}\sum_{k=1}^{2}(S_{H}^{k}(t)+R_{H}^{k}(t))},$$

using the bounds of the control, we obtain the characterisation given in Equation (31).

### 3.4 Numerical simulations

In this section we present a numerical simulation of the model. In our numerical simulation, we assume that around 83% of the population has at least one dengue serotype as found in Indonesia [35]. The initial conditions are $S_{H}(0)=0.15$, $E_{H}^{1}(0)=E_{H}^{2}(0)=0$, $I_{H}^{1}(0)=I_{H}^{2}(0)=0.01$, $R_{H}^{1}(0)=R_{H}^{2}(0)=0.415$, $A_{N}(0)=0.791111869731644$, $S_{N}(0)=1.031725009589116$ and the other mosquito populations are zero. For control, we use cost values are follows. $W_{1}=W_{2}=216.5$, $W_{3}=W_{4}=433$, $W_{5}=W_{6}=2.27$. The values are direct and indirect cost of hospitalised and ambulatory dengue cases, vaccine delivery and cost to obtain vaccine dose in Indonesia [36]. The direct cost represents the cost associated with resource utilization. Indirect cost has associated with the opportunity cost of time required to obtained vaccine dose and social perspective about the disease [36], [37], [38].

**Figure 7:**
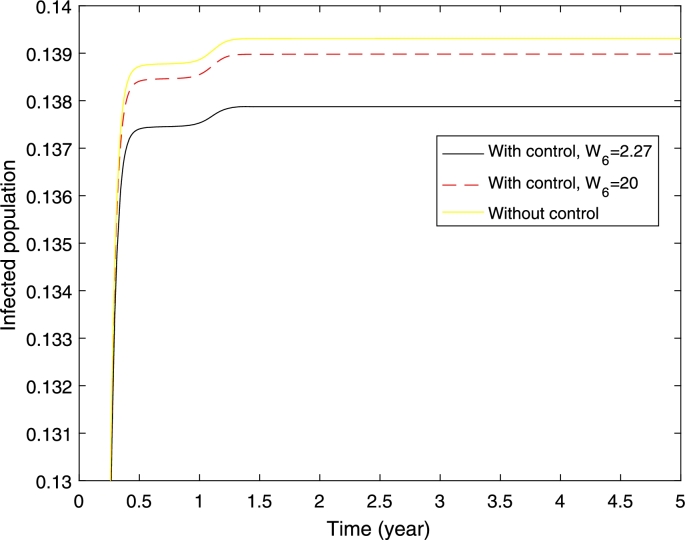
Numerical simulations with and without control when only seropositive individuals are vaccinated. The cost of vaccination (*W*_6_) are 2.27 and 20 as given in legend, *W*_1_ = *W*_2_ = 380. The initial conditions are given in the text.

**Figure 8:**
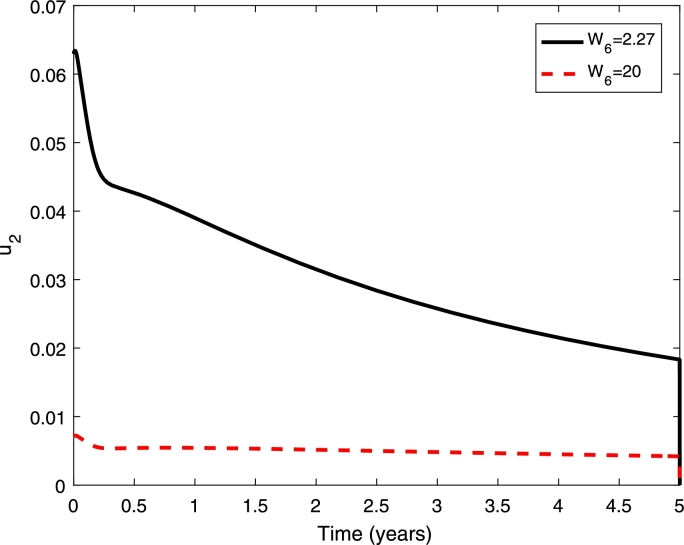
Control profiles with different cost of vaccination *W*_6_ = 2.27 and 20 when only seropositive individuals are vaccinated.

Figs. 7 presents the proportion of dengue cases with and without control/vaccination when we only vaccinate seropositive individuals. It shows that the proportion of dengue cases decreases with implementation of vaccination and higher decreases has been obtained if the vaccination cost is cheaper. When the vaccination cost is cheaper, the vaccination rate is higher (see Fig. 8).

**Figure 9:**
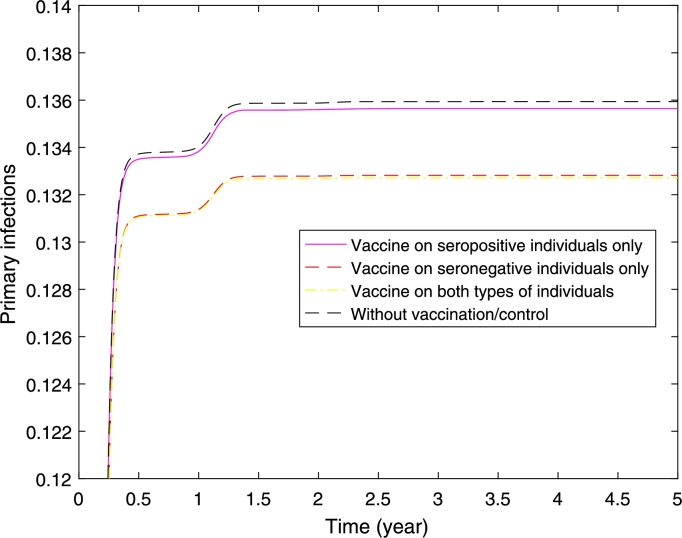
Primary infections where half of the populations are seropositive and the other half are seronegative. It presents three scenarios of implementation: vaccine on seropositive individuals only, seronegative individuals only, and both seropositive and seronegative individuals.

**Figure 10:**
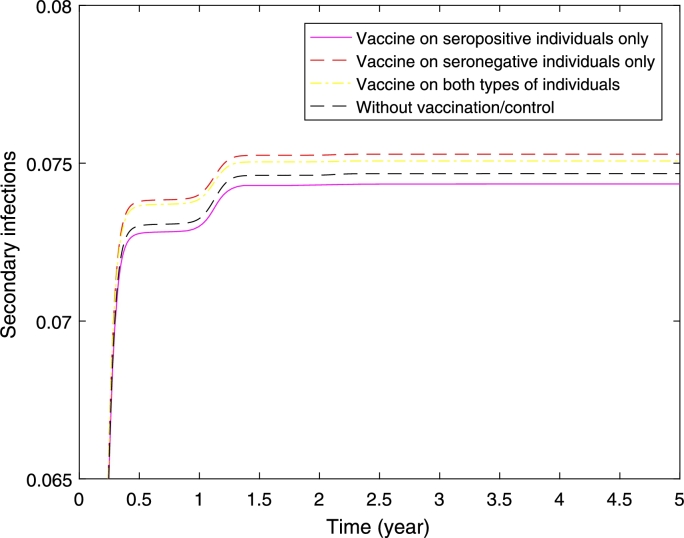
Secondary infections where half of the populations are seropositive and the other half are seronegative. It presents three scenarios of implementation: vaccine on seropositive individuals only, seronegative individuals only, and both seropositive and seronegative individuals.

Figs. 9 and 10 presents the case where the half of the population are seronegative and the other half are seropositive, and the vaccination is implemented on seronegative only, seropositive only, and both types of individuals. Figs. 9 shows that higher reduction in the proportion of primary dengue infections can be obtained if we vaccinate seronegative individuals only or both types of individuals. The result shows similar reduction in the proportion of primary dengue infections when we vaccinate seronegative individuals only or both types of individuals. This implies that it is sufficient to vaccinate seronegative individuals only to obtain higher reduction in the proportion of primary infections.

**Figure 11:**
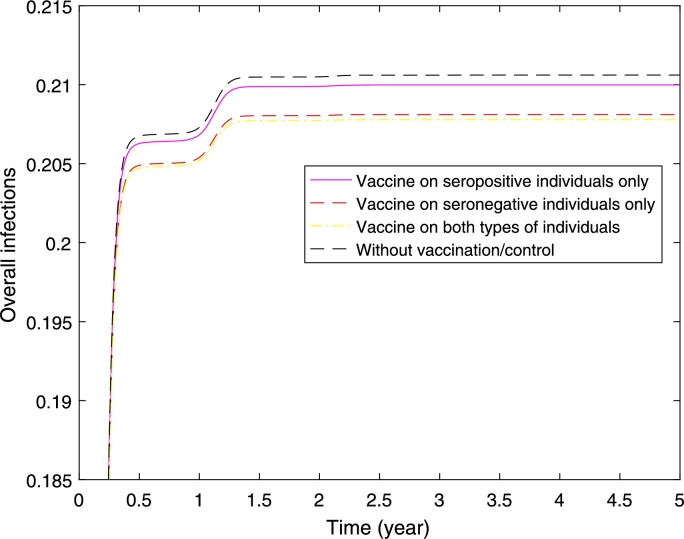
Overall dengue infections where half of the populations are seropositive and the other half are seronegative. It presents three scenarios of implementation: vaccine on seropositive individuals only, seronegative individuals only, and both seropositive and seronegative individuals.

**Figure 12:**
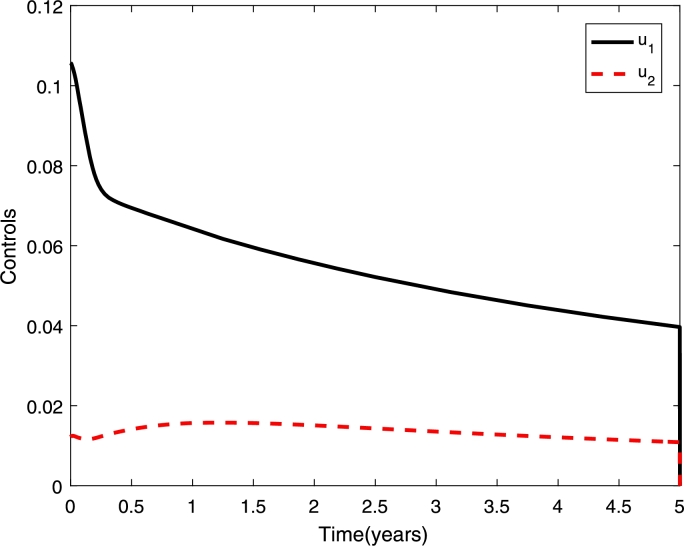
Control profiles for vaccinations on seronegative (*u*_1_) and seropositive (*u*_2_) individuals, when we vaccinate both types (seronegative and seropositive) of individuals.

Fig. 10 shows higher reduction in the proportion of secondary infections if we vaccinate seropositive individuals only. Interestingly if we vaccinate the seronegative individuals only, or both types of individuals the proportion of secondary infections is higher compared to without vaccination. Although the proportion of secondary infections is high when we vaccinate seronegative individuals only or both types of individuals, a higher reduction in the proportion of overall dengue cases has been obtained (see Fig. 11). Furthermore, the control profile (see Fig. 12) shows higher control rate on seronegative individuals.

## 4 Discussion and conclusions

In this paper, we develop a dengue mathematical model with vaccination: single and two serotype dengue model. An optimal control approach is used to assess the optimal vaccination strategy against dengue. We parameterise the model against 2016 dengue data in Kupang city, East Nusa Tenggara, Indonesia. We investigate the effects of vaccination on seronegative, seropositive individuals only and both types of individuals. In optimal control approach, we use quadratic terms in the control variables to capture the nonlinear cost in the implementation of controls. This approach is rather conventional and has been frequently used in the epidemiological modelling including dengue modelling [11], [13], [26], [15], [23], [39], [40], [12]. Rawson et al. implemented optimal control approach to investigate the effects of vector control and vaccination on dengue transmission dynamics. They used quadratic terms in the control variables as to represent the nonlinear cost [23], which has been also used in the other work for the similar reason to investigate the dengue transmission dynamics in the presence of controls [11], [13], [26], [15], [23], [39], [40], [12].

Sensitivity analysis of the single serotype model shows that the transmission probability $\left(T_{N}\right)$, the biting rate $\left(b_{N}\right)$, and the vaccination rate (*u*) are the most influential parameters. It shows that the mosquito's related parameters do not have much impact on the reduction of dengue cases when the vaccination is implemented. This is interesting since in the absence of vaccination, the mosquito-related parameters are also the influential parameters [41]. This may imply that implementing vaccination may be sufficient in reducing the proportion of dengue cases. However, further work needs to be conducted to understand the vaccine delivery. For the two-serotype dengue model, the results are similar where the transmission probability $\left(T_{N}\right)$, the biting rate $\left(b_{N}\right)$, and the vaccination rate on seronegative individuals $\left(u_{1}\right)$ and the average death rate ($\mu_{N0}$) are the influential parameters. This implies that controlling these parameters are sufficient to reduce the proportion of overall transmission. Furthermore, these parameters are influential since the early period of epidemics and remain influential until the end of period.

With the implementation of vaccination, a reduction in the proportion of dengue cases can be obtained. Furthermore, if the vaccination is implemented in seronegative individuals only, it results in higher proportion of dengue secondary infections. Our results showed that an increase in the secondary infections can be obtained if we vaccinate seronegative individuals only. To reduce the risk of obtaining the more dangerous forms of dengue, it is better to vaccinate seropositive individuals. Aquiar et al. [6] also found that if we vaccinate seropositive individuals, a higher reduction in the hospitalized case can be obtained. In the case of Indonesia, in particular Kupang, where the majority of individuals (around 83% [35]) have been exposed to at least one dengue strain, the vaccination program can be implemented and it has possibility to reduce the proportion of dengue cases. In addition, when the meantime of human turnover rate is faster, the outbreak would happen starting from around year 58. If the constant vaccination is implemented in the first five years, the time at which outbreaks occurs has shifted around 20 years (results not shown here). This phenomena cannot be seen in the model without seasonality. Further exploration of this is required, which is the subject of future work. Future research also considers the reinfection with the same serotype which may happen as found by Anggriani et al. [42] or combination of vaccination and *Wolbachia* intervention [43]. This may complicate the dynamics of dengue transmission under vaccination strategy. In addition, in this paper, we use quadratic terms in the control variables, which is common in optimal control of epidemiological models. The use of other terms in control variables should be considered for future investigation, which can provide additional insights on dengue transmission dynamics in the presence of controls.

## Declarations

### Author contribution statement

M. Ndii: Conceived and designed the experiments; Performed the experiments; Analyzed and interpreted the data; Contributed reagents, materials, analysis tools or data; Wrote the paper.

A.R. Mage: Performed the experiments; Analyzed and interpreted the data.

J.J. Messakh: Analyzed and interpreted the data; Contributed reagents, materials, analysis tools or data.

B.S. Djahi: Performed the experiments.

### Funding statement

This research has been funded by 10.13039/501100009509Ministry of Research, Technology and Higher Education, Indonesian Government through Fundamental Research scheme 2019-2021 (No: 48/UN15.1.19/PL/2019).

### Declaration of interests statement

The authors declare no conflict of interest.

### Additional information

No additional information is available for this paper.
